# Middle-Georgia Medical Society

**Published:** 1872-06

**Authors:** T. R. Kendall, C. S. Strother


### MIDDLE-GEORGIA MEDICAL SOCIETY.

The regular bi-monthly meeting of the Middle-Georgia Medical Society was held in Thomaston, Georgia, Wednesday, May 15th, 1872—Vice-President Dr. T. R. Kendall in the chair.

The roll was called, and the minutes of the previous meeting read and adroped.

Drs. Drake and Gardener made application for active membership, which was referred to the Committee on Credentials, composed of Drs. Perdue and Strother.

The names of Drs. Rogers and White were proposed as honorary members, which was unanimously acquiesced in by the Society.

Dr. J. E. Cook, of Culloden, Georgia, read a paper, under the Section of Anatomy.

Dr. Kendall made some pertinent remarks, under the same head, respecting the intimate relation which anatomy bears to the remaining branches of the profession.

Under the Section of Materia Medica, Dr. Strother read a paper on the action of carbolic acid in menorrhagia and metrorrhagia, reporting several cases.

Dr. Farmer made a verbal report of two cases of obstinate menorrhagia, treated as above, at the suggestion of Dr. Strother, at a late interview.

Dr. Farmer also read a very interesting paper on morphia as a preventive of the hot stage of intermittent fever, when given in one-third of a grain doses, just as the cold stage is passing off. The Doctor had never seen it fail to prevent the hot stage.

Drs. Rogers and Kendall discussed the merits of carbolic acid as an external application, but more particularly in regard to its efficacy in aborting the pustules of small-pox.

Dr. Leseur reported a case of morbid anatomy, observed in a recent post-mortem.

Dr. Perdue reported a case of aneurism of the abdominal aorta, which he considered he was conducting to convalescence by appropriate remedies.

Dr. Strother read a report of a case of true croup, treated by means of the steam atomizer.

Dr. C. S. Leseur then delivered an appropriate address on medical ethics and kindred subjects.

*Resolved,* That the members of the Society be requested not only to report facts in connection with their special Sections, but also cases of interest in general practice.

*Resolved,* That the minutes of this meeting be published in the ATLANTA MEDICAL AND SURGICAL JOURNAL.

On motion, the Society adjourned to meet in Culloden, Georgia, on the third Wednesday in July next (July 17, 1872.)

T. R. KENDALL, M.D., First Vice-President.

C. S. STROTHER, M.D., Corresponding Secretary.

